# Purification of Aqueous Media by Biochars: Feedstock Type Effect on Silver Nanoparticles Removal

**DOI:** 10.3390/molecules25122930

**Published:** 2020-06-25

**Authors:** Agnieszka Tomczyk, Katarzyna Szewczuk-Karpisz, Zofia Sokołowska, Milena Kercheva, Emil Dimitrov

**Affiliations:** 1 Institute of Agrophysics, Polish Academy of Sciences, Doświadczalna 4, 20-290 Lublin, Poland; k.szewczuk-karpisz@ipan.lublin.pl (K.S.-K.); z.sokolowska@ipan.lublin.pl (Z.S.); 2 Institute of Soil Science, Agrotechnology and Plant Protection “N. Poushkarov”, Shosse Bankya 7, Sofia 1080, Bulgaria; mkercheva@abv.bg (M.K.); e.dimitrov7@gmail.com (E.D.)

**Keywords:** biochar, metal nanoparticles, aqueous purification media

## Abstract

Due to the harmful effects of nanoparticles in the environment, their effective removal from aqueous media is of great importance. This paper described the research on the silver nanoparticles (Ag-NPs) sorption on biochars obtained from different feedstock types. The sorbents were produced through pyrolysis (double-barrel method) of the vineyard (BV), paulownia tree (BP), and tobacco (BT). BV exhibited the highest specific surface area, porosity, value of variable surface charge, and content of surface acidic functional groups among the used biochars. The pseudo-second order model best described the obtained adsorption kinetics, whereas the Freundlich model accounted for the registered adsorption data. The Ag-NPs removal was highly efficient in the case of BV, especially in the nanoparticle concentration range 50–500 mg/L. Thus, this biochar can be considered as an ecofriendly, effective, low-cost organic adsorbent, potentially used in the aqueous media purification.

## 1. Introduction

Currently, in the 21st century, the use of silver nanoparticles (Ag-NPs) is becoming more and more common. These compounds are used in agriculture, medicine, textiles, cosmetics, pharmaceuticals, dentistry, cleaning products, etc., [1] owing to their antibacterial activity against Gram-negative and Gram-positive bacteria. Forecasts predict that the production of Ag-NPs may reach over 58.000 tons annually between 2011 and 2020 [2]. However, it is also pointed out that the excessive use of Ag-NPs and their appearance in the environment may have a harmful impact on the quality of the environment and the health of organisms [3]. Ag-NPs are treated as a new type of environmental pollution excessively released from consumer products [4]. Ag-NPs, present in textiles, cosmetics or medicines, are released into surface waters and groundwater [5]. Then, they can also reach the soil and organisms living in it [6]. Unfortunately, the modern wastewater treatment systems do not completely remove nanoparticles due to their small size [7]. The regulations regarding water quality describe only acceptable concentrations of elementary metals, such as copper, silver, zinc or titanium in surface, ground, and drinking water, and do not include nanoforms of these elements. Ag-NP analogs of these macroscopic toxic metals may have similar toxicity to the ionic form [4] and threaten aquatic and soil organisms as well as people [8]. The small size and reactivity of nanoparticles enable their penetration into tissues and interference with biochemical processes [9]. The behavior of nanoparticles in the environment depends mainly on conditions such as pH, temperature, and the presence of other chemical compounds [10]. Therefore, these problems should be tested to prevent excessive amounts of nanoparticles in the environment.

Many processes have been proposed for the removal of Ag-NPs from water: adsorption, aeration, and coagulation [11]. Unfortunately, many of them are expensive and inefficient. Adsorption can serve as an alternative. Some natural and synthetic adsorbents for Ag-NP removal have been investigated, e.g., activated carbon [12], dolomite limestone [13], activated sludge [14], natural clinoptilolite [15], nanofiber membrane [16], poly(vinyl alcohol) [17], and ZnO [18]. Biochars can be also promising materials in the removal of nano impurities [10,19]. These solids are inexpensive, economical, and eco-friendly adsorbents.

Biochar is a carbon-rich material formed as a result of biomass pyrolysis [20]. It can be used not only as a renewable fuel, but also as an addition improving aqueous media [21]. Biochar exhibits a high carbon content as well as a substantial concentration of micro- and macro-elements (including potassium, sodium, magnesium, and calcium). This material is generally characterized by a high specific surface area and a high content of surface functional groups [19,22,23,24]. Hernandez-Mena et al. [24] showed that biochar has a high porosity, with longitudinal pores with sizes from micro- to macro-pores.

In the literature, there are many articles discussing the negative effects of nanoparticles on natural environments and living organisms. Numerous alarming data were published, and therefore the investigation of a protection method against nanoparticles is of great importance. It must also be emphasized that no comprehensive solution has been proposed yet. Due to the fact that there are no reports in the literature describing Ag-NP separation from aqueous solutions by natural organic adsorbents, this paper was aimed at the efficiency of metallic nanoparticle (Ag-NP) removal by biochars. In this way, the study adopted a comprehensive, innovative approach to the above issue. The performed experiments included the following three steps: (i) study of the impact of various kinds of feedstock on the structure of biochar, (ii) study of the adsorption kinetics and equilibrium isotherm of adsorption Ag-NPs on biochars, (iii) evaluation of the efficiency of biochars in Ag-NP removal from aqueous media.

## 2. Results and Discussion

### 2.1. Characteristic of Ag-NPs

**Figure 1:**
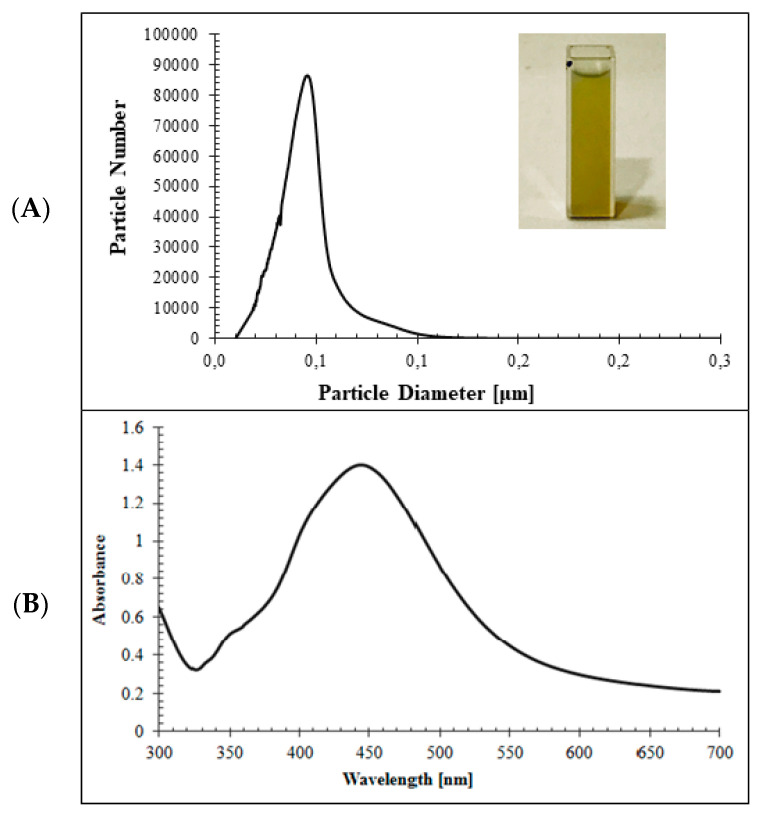
Characteristics of Ag-NPs: (**A**) particle number (in billions) vs. particle diameter and (**B)** UV–VIS absorption spectrum.

The results presented in Figure 1 show characteristics of Ag-NPs obtained by the chemical reduction method.

The synthesized Ag-NPs (Figure 1) were obtained as a green colloid solution and were characterized by UV–VIS spectrophotometric analysis in Figure 1B, and showed a maximum peak of absorbance at 440 nm. The results of the CPS analysis of the Ag-NP solution are presented in Figure 1A. They showed that the particles of diameters equal to 46 nm were the most numerous in the examined sample. The shape of the Ag-NPs was cube [25]. The Ag-NPs did not form aggregates (also after 70 min corresponding with the adsorption time). The nanoparticle solution was stable for 4 days (after this time silver precipitated).

### 2.2. Biochar Characteristics

The experiments included three discrete biochars: vineyard (BV), paulownia tree (BP), and tobacco (BT). The results presented in Table 1 as well as in Figure 4 showed clear differences among biochars obtained from different biomasses.

Biochar samples exhibited a relatively high variable surface charge *Q*. BV was characterized by the highest *Q* parameter, while BT by the lowest. *Q* provides information about the presence of surface functional groups: carboxylic, phenolic, and lactonic groups. This was confirmed by Boehm’s method. BV exhibited the highest content of surface acidic functional groups, and BT the lowest. This effect was attributed to the content of lignin or cellulose in the biomass [26].

The obtained results also indicated that the biochars BV and BP had similar textural properties. They are characterized by a high content of pores of an average diameter of about 2 nm and a relatively high specific surface area equal to 98.9 and 83.9 m^2^/g for BV and BP, respectively. The total pore volume is 0.04 for the BV and 0.03 cm^3^/g for the BP. On the other hand, the BT has poor porosity parameters. It contains large mesopores of an average diameter of 19.9 nm. Moreover, its *S_BET_* parameter equals 1.9 m^2^/g, whereas its *V_t_* parameter equals only 0.01 cm^3^/g. This is dictated by different thermal degradations of biomass. During pyrolysis, volatile matter is released to varying degrees, which creates differences in micropore contribution and pore volume, as well as specific surface area [26].

**Figure 2:**
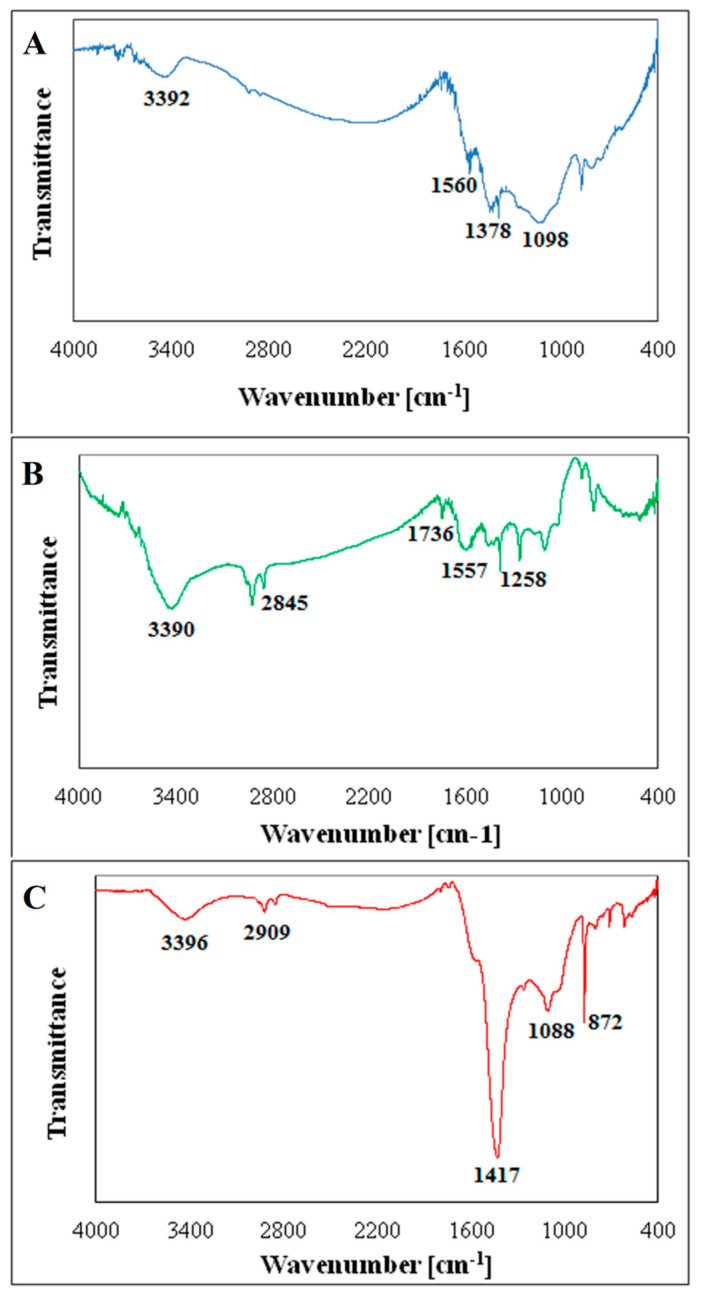
FTIR spectra of biochars: (**A**) vineyard (BV); (**B**) paulownia tree (BP), and (**C**) tobacco (BT).

Experimental data obtained from the FTIR analysis are presented in Figure 2.

All spectra contained specific bands corresponding with O–H groups stretching (~3390–3430 cm^−1^), methyl C–H groups stretching (~2910 cm^−1^), methylene C–H groups stretching (~2850 cm^−1^), deformation of C−H bond in aliphatic and alicyclic systems (~1380 and 1420 cm^−1^), the stretching of C–O–C and –OH groups of esters associated with the degradation of cellulose and hemicelluloses (~1080–1100 cm^−1^), and the stretching of C–H groups in aromatic structures (~800–880 cm^−1^). Additionally, the spectra of BV and BP contained the bands that can be attributed to the vibrations of C–O bonds in carboxylic and lactone groups as well as the stretching of C–C groups present in aromatic rings (~1560 and 1740 cm^−1^). The spectrum of BP was also characterized by the band corresponding with the C–O–C stretching of aryl ethers, as well as phenolic groups associated with the degradation of lignin (~1260 cm^−1^). The bands occurring around ~620–720 cm^−1^, visible on every spectrum, may be associated with mineral matter.

Many studies confirmed that the content of surface acidic functional groups, as well as the value of *S_BET_* of biochars, influence their adsorption capacity [27]. For example, Tomczyk et al. [28] showed that wood biochar had a large adsorption capacity relative to Cu, which was associated with a high value of *S_BET_*. Wang et al. [29] and Sumaraj et al. [30] reported that surface functional groups of biochar: –COOH, –OH, and –COR, had a high impact on the heavy metal adsorption level.

### 2.3. Kinetics of Ag-NPs Adsorption on Biochar

**Figure 3:**
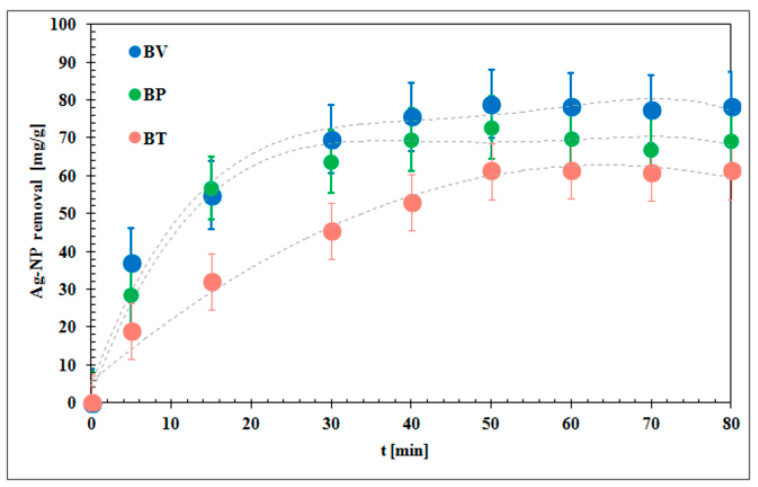
Pseudo-second order adsorption kinetics of Ag-NPs on biochars.

The experimental Ag-NP adsorption kinetics with the fits obtained from a pseudo-second order equation are presented in Figure 3. The results showed that the time of 70 min was sufficient to reach equilibrium. This time was used in the measurements of Ag-NP adsorption isotherms. The kinetic parameters, presented in Table 2, showed clear differences among experimental biochars.

Experimental kinetic isotherms were fitted to the two following theoretical adsorption models: the pseudo-first order model (Equation (2)) and the pseudo-second order model (Equation (3)). The pseudo-second-order model matched better experimental data (the squared correlation coefficient R^2^ was higher than 0.98) compared to the pseudo-first-order model (Table 2). Ruíz-Baltazar et al. [15] reported that the experimental data of Ag-NPs adsorbed on the natural clinoptilolite fitted better with the pseudo-second-order model. Furthermore, Dhandayuthapani et al. [17] observed that the pseudo-second-order model has the best fit to a series of Ag-NP adsorption on poly(vinyl) alcohol data.

Ho and McKay [31] reported that the high value of the R^2^ suggests multiple adsorption kinetic mechanisms, i.e., surface binding interactions that precede chemisorption and precipitation. Based on the obtained rate constants, it was found that the fastest adsorption occurred on BV *k*_2_ = 0.44 × 10^−2^ g/mg·min and the slowest on BT *k*_2_ = 0.1 × 10^−2^ g/mg·min. Similar observations were reported by Banach et al. [13] which proved that Ag-NP adsorption on dolomite limestone was fast (0.15 × 10^−2^ g/mg·min).

The adsorption capacity of BV (88.9 mg/g) was the highest among all biochars (75.2 mg/g for BP and 72.5 mg/g for BT). It has been noted in previous studies that adsorption capacity depends on the kind of adsorbent. These parameters equaled 8.38 × 10^−4^ mg/g for natural clinoptilolite [15], 47.56 mg/g for ZnO [18], and 143.40 mg/g for nanofiber membrane [16].

### 2.4. Equilibrium Adsorption of Ag-NPs on Biochar

**Figure 4:**
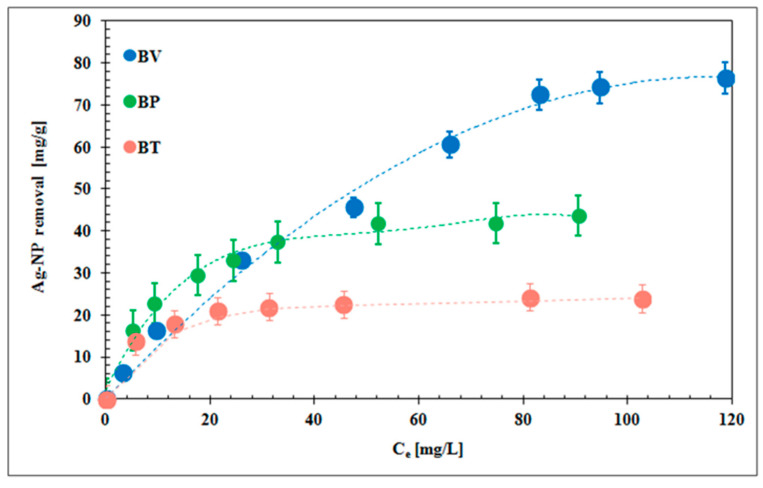
Freundlich adsorption isotherms of Ag-NPs on biochars.

Figure 4 presents plots that compare experimental data with the fits obtained from the theoretical Freundlich model.

The parameters calculated from the selected models were summarized in Table 3. The coefficients of R^2^ were determined using linear regression.

By analyzing the theoretical and experimental points, it was concluded that Freundlich isotherms best describe silver nanoparticle adsorption on all biochars. The Freundlich model yielded the squared correlation coefficient R^2^ > 0.97, and the Langmuir model produced R^2^ > 0.69. The values of 1/*n* were, respectively, equal to 0.41 for BV, 0.72 for BP, and 0.92 for BT. The parameter 1/*n* was below 1, which proved that the surfaces of the experimental biochars were heterogeneous. The lowest 1/*n* value indicated that BV possessed the most heterogeneous surface. The calculated Freundlich constant K_F_ was 0.46 [mg/g(L/mg]^1/*n*^ for BV, 1.69·10^−6^ for BP, and 6.01·10^−9^ for BT. Banach et al. [13] observed that the adsorption of Ag-NPs on dolomite limestone also fitted better to the Freundlich model. Similar conclusions were formulated during the study on Ag-NP adsorption on activated carbon. In that case, it was reported that 1/*n* was below 1 (0.75) and *K_F_* was 1.8 [mg/g(L/mg]^1/*n*^ [12].

It should also be mentioned that the Ag-NP adsorption on BP and BT reached the equilibrium for lower concentrations than on BV. The isotherm curve for BP and BT achieved equilibrium for initial Ag-NP concentrations equal to and greater than 50 mg/L. In turn, in the case of BV the equilibrium state was reached for the initial Ag-NP concentrations equal to and higher than 350 mg/L. This difference was probably dictated by the most heterogeneous surface of BV. Among tested solids, the vineyard biochar was characterized by the highest variable surface charge, the largest specific surface area, and the largest micropore volume. This biochar adsorbs the largest amount of nanoparticles from aqueous solution.

The values of Ag-NP removal efficiency for the initial Ag-NP concentration 500 ppm, calculated from experimental data, were 95.2% for BV, 51.7% for BP, and 34.7% for BT. This indicated that 24.1 mg/L (4.8%) of nanoparticles in the case of BV, 241.6 mg/L (48.3%) in the case of BP, and 326.7 (65.3%) in the case of BT remained in the solution. In turn, the Ag-NPs removal efficiency for the initial Ag-NP concentration 50 ppm, calculated from experimental data, were 57.2% for BV, 50.7% for BP, and 27.5% for BT. In these systems 21.4 mg/L (42.8%, BV), 24.6 mg/L (49.3%, BP), and 36.25 mg/L (72.5%) of nanoparticles remained in the solution. The chemical character of the Ag-NP adsorption process on the biochar surface is dictated by the hydrogen bond formation between oxygen atoms within acidic residues of adsorbent and –OH groups of Ag-NPs [10]. The hydroxyl groups are present in the structure of ascorbic acid stabilizing nanoparticles. These moieties are the most protruding groups of Ag-NPs and act as proton donors. In turn, oxygen atoms of biochar surface groups are proton acceptors. Uchimiya et al. [10] observed that hydrogen bonds are also created between a gold nanoparticle-bound citrate capping agent and surface carboxyl groups present on biochar produced at 300 °C. Furthermore, Banach et al. [13] reported that Ag-NPs were also adsorbed on dolomite limestone by hydrogen bonds.

**Figure 5:**
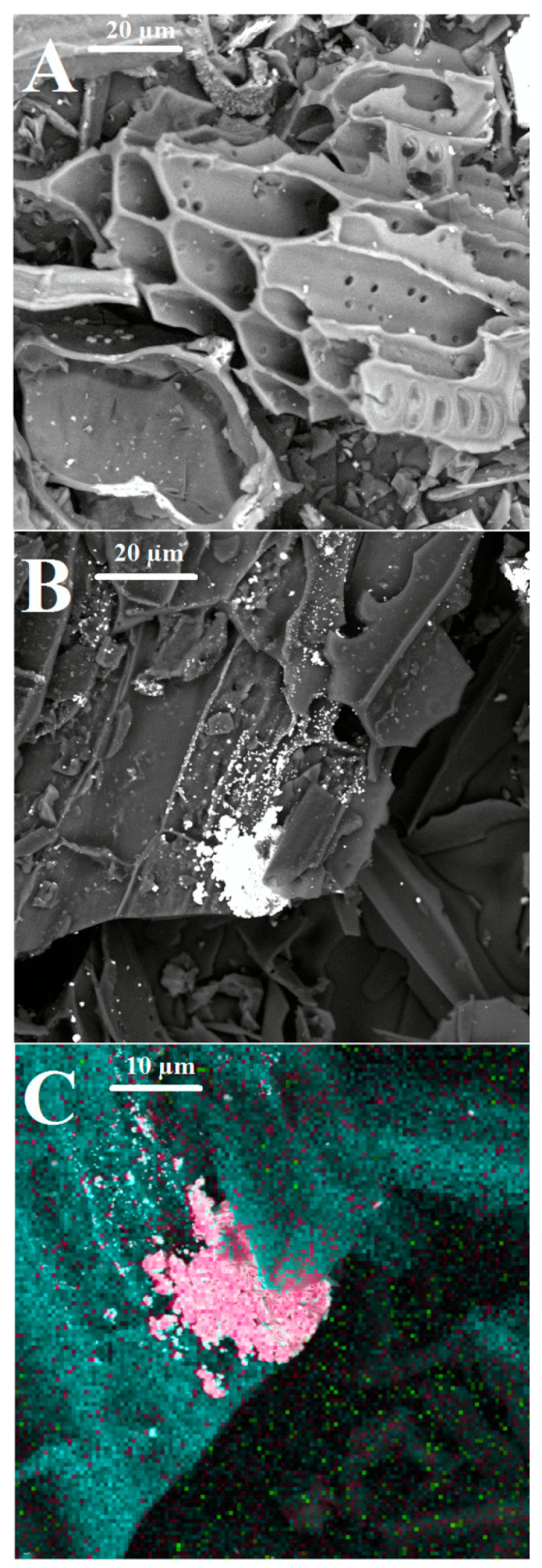
The SEM images of BV before (**A**) and after (**B**) adsorption (white color: Ag-NPs) and the results of EDS analysis (**C**) of the selected biochar after adsorption (blue color: carbon, green color: oxygen, pink color: silver).

Based on the results of the performed adsorption study, it can be stated that biochar obtained by vineyard pyrolysis is the best adsorbent of Ag-NPs in their concentration range 50–500 mg/L. This biochar may be considered as a potential, eco-friendly adsorbent used in water purification. BV has the highest content of acidic functional groups (carboxylic and phenolic ones) and well-developed specific surface area. Due to these facts, it has the greatest affinity to create hydrogen bonds with Ag-NP. Its adsorption capacity relative to silver nanoparticles is the highest (among all tested solids). The high adsorption level on the vineyard biochar surface was also confirmed by SEM observations and EDS analysis. The obtained images are presented in Figure 5.

These showed the surface morphology of biochar before and after the Ag-NP adsorption. The SEM image of BV showed that biochar had a heterogeneous surface. The white and pink points were attributed to Ag-NPs adsorbed on BV. The BV surface was partially covered with Ag-NPs. Adsorbed nanoparticles may have formed clusters on the solid. Besides silver nanoparticles, biochar was also used in the removal of other contaminants, e.g., heavy metals [28,32,33], dyes [34,35], gold nanoparticles [10], antibiotics [36,37], and PAH [38,39].

## 3. Materials and Methods

The biochars were produced during the biomass pyrolysis process, which is called the “double barrel method” [40]. The temperature reached in the kiln was not measured in our study. Deal et al. [40] estimated that the highest temperature reached inside such a double-barrel kiln by slow pyrolysis was between 400 and 600 °C at the top and between 600 and 800 °C at the bottom. At the beginning, biochars were air-dried and sieved through 2 mm meshes.

### 3.1. Specific Surface Area

Textural properties of the used biochars were determined using low-temperature nitrogen adsorption-desorption isotherms method (sorptometer ASAP 2420, Micrometrics, Norcross, GA, USA). Before the measurement, the solids were degassed at 200 °C under vacuum. Specific surface area (*S_BET_*) and pore size distribution (PSD) of the samples were calculated using BET and BJH methods. The total pore volume (*V_t_*) was established at a relative pressure *p*/*p*_0_ = 0.99.

### 3.2. Ag-NPs Synthesis

The silver nanoparticles (Ag-NPs) were obtained by the chemical reduction method [25]. Silver nitrate (AgNO_3_) was used as a silver nanoparticle precursor. Ascorbic acid was used as a reducing agent, whereas polyvinyl pyrrolidone (PVP) was used as a stabilizer. For the preparation of aqueous solutions, deionized water was used. A total of 75 mg of AgNO_3_ was added into 150 mL of deionized water containing 5 g of PVP and such prepared solution was heated to 100 °C. Then, the aqueous solution of ascorbic acid in the molecular ratio AgNO_3_/C_6_H_8_O_6_ equal to 1.2 was added dropwise into the silver nitrate solution and stirred for 1 h. The above method allowed the Ag-NP solution with a concentration of 500 mg/L to be obtained. UV-VIS spectra were recorded with a 1 cm path length quartz cell at a resolution of 1 nm from 300–700 using a Jasco V-530 UV/VIS Spectrophotometer (Tokyo, Japan). Deionized water was used as the reference sample to take the blank spectrum for all measurements.

### 3.3. The Average Size of Ag-NPs

The average size of Ag-NPs was determined using a CPS analyzer (CPS Instruments, Anaheim, CA, USA). In this method, the particle diameter is calculated by the software based on the changes in the light absorption during the measurement. The disc rate was set to the value of 22,000 rpm. The sucrose solutions with the concentrations of 8 and 24% were used in the gradient preparation. The particle diameter was measured in the range of 10–250 nm. The Ag-NP solution used in the study had the concentration of 500 ppm.

### 3.4. Quantitative Determination of Surface Functional Groups

The determination of surface oxygen groups was carried out using the Boehm titration [41]. This method is based on the assumption that the acidic constants of carboxylic, lactonic, and phenolic groups differ by several orders of magnitude, and therefore it is possible to neutralize these groups with properly selected reagents. The acidity of individual functional groups depends on their location and environment, i.e., on the size of the layers and the type or position of the other substituent.

### 3.5. Determination of the Distribution of the Surface Negative Charge and Dissociation Constants of Functional Groups

The measurements were made using a Titrino 702 SM (Metrohm, Herisau, Switzerland), an automatic titrator. Based on the potentiometric data obtained in the experiment, variable surface charge *Q* was calculated [42].

### 3.6. Qualitative Determination of Surface Functional Groups—FTIR Spectroscopy

The chemical character of the biochar surface was determined using Fourier Transform Infrared Spectroscopy (FTIR). The spectra were obtained using Nicolet 8700A FTIR spectrometer (Thermo Scientific, Somerset, NJ, USA). The samples were prepared in the form of pellets with KBr.

### 3.7. SEM Analysis

Scanning electron microscopy (Phenom ProX, Thermo Fisher Instruments, Somerset, NJ, USA) was used to examine surface morphology of biochar before and after the adsorption process. The presence of Ag-NPs on the biochar surface was confirmed by EDS analysis (energy-dispersive X-ray spectroscopy).

### 3.8. Batch Adsorption Experiment

A total of 40 mg of biochar was added to 8 mL of the Ag-NP solutions (500 mg/L for kinetics and 0, 50, 100, 150, 200, 250, 350, 400, and 500 mg/L for equilibrium measurements). The suspensions were permanently mixed (about 5, 15, 30, 40, 50, 60, 70, and 80 min for kinetics and about 70 min for equilibrium adsorption) with rotators under constant pH conditions (5.0 ± 0.1), at room temperature (19 ± 2 °C). After completion of Ag-NP adsorption on biochar, the solutions were filtered, and the concentration was measured using a UV/VIS spectrophotometer at λ = 440 nm. The process was repeated three times for all samples, and the graphing points were obtained from the averages.

The Ag-NPs adsorbed amount (mg/g) was calculated as follows:

(1) $$q_{e}=\frac{\left(C_{0}-Ce\right)\cdot V}{m},$$

where *C*_0_ is the initial concentration of heavy metal (mg/L), *C_e_* is the heavy metal concentration at equilibrium (mg/L), *m* is the mass of the sample (mg), and *V* is the volume of the solution (L).

The pseudo-first order [43], used in Equation (2), and the pseudo-second order [31], used in Equation (3), were selected among different models that can be applied to describe the adsorption kinetics. These equations are represented by the following linearized equations:

(2) $$\ln\left(q_{e}-q_{t}\right)=\ln q_{e}-k_{1}\cdot t,$$

(3) $$\frac{t}{q_{t}}=\frac{1}{k_{2}\cdot q_{e}^{2}}+\frac{t}{q_{e}},$$

where *q_t_* (mg/g) is the Ag-NP removal capacity at time *t* (min), *q_e_* (mg/g) is the Ag-NP removal capacity at equilibrium, and *k*_1_ (1/min) and *k*_2_ (g/mg·min) are the reaction rate constants.

Two of the most common equations in the literature (Freundlich and Langmuir isotherms) were used to describe adsorption processes. The Freundlich isotherm was one of the simplest, originally introduced in the study of adsorption on animal charcoal [44]. Sips [45] proved that the Freundlich isotherm can describe adsorption on energetically heterogeneous surfaces that are characterized by an exponential distribution of the adsorption sites with energy.

The Freundlich isotherm is expressed as follows:

(4) $$q_{e}=K_{F}{\left[\frac{C_{e}}{a_{m}}\right]}^{n},$$

where *q_e_* is the amount of adsorbed Ag-NPs at equilibrium (mg/g), *C_e_* is the equilibrium concentration of adsorbate in the solution (mg/L), and *K_F_* (in units of *q_e_*) and *n* (0 < *n* < 1) are the Freundlich constants (mg/g(L/mg)^1/*n*^), which represent the adsorption capacity and the “heterogeneity parameter”, respectively. The parameter *a_m_* is a formal parameter equal to the unity in the units of *C_e_* that was introduced in order to display correct units on both sides of Equation (4).

Langmuir isotherm [46] is expressed as follows:

(5) $$q_{e}=\frac{Q_{m}K_{L}C_{e}}{1+K_{L}C_{e}},$$

where *Q_m_* is the maximum number of Ag-NPs in the monomolecular layer (mg/g) and *K_L_* is the Langmuir constant related to the affinity of the adsorbate for active sites (L/mg). This equation can be used if the adsorption process does not lead to the development of multilayers, or does not occur in pores or with capillary condensation.

The efficiency, *E* (%), of Ag-NP removal can be expressed as follows:

(6) $$E\%=\frac{C_{A}}{C_{0}}\cdot 100\%,$$

where *C_A_* is the concentration of sorbed nanoparticles (mg/L) and *C*_0_ is the initial concentration (mg/L).

## 4. Conclusions

This research investigated the ability of biochars to adsorb Ag-NPs in aqueous media. The feedstock type influence on the textural and surface biochar characteristics, such as the specific surface area, porosity, variable surface charges, and the content of functional surface acidic groups, was also determined. The obtained results showed that BV exhibited the highest specific surface area, porosity, variable surface charge, and content of surface acidic functional groups among all tested biochars. The adsorption kinetics of Ag-NPs on biochars were well-described by the pseudo-second-order model, whereas Ag-NP adsorption isotherms by the Freundlich model. The correlation coefficient was above 0.97. The Ag-NP adsorption capacities were 88.9 mg/g for BV, 75.2 mg/g for BP, and 72.5 mg/g for BT. Ag-NP removal efficiency was the highest for the biochar produced from the vineyard (95.2%). Therefore, the selected biochar is an efficient adsorbent of silver nanoparticles in their concentration range of 50–500 mg/L and may be considered as an eco-friendly material used in water purification. The examined adsorption was probably based on the hydrogen bond creation between oxygen atoms present on the biochar surface and hydroxyl groups of ascorbic acid stabilizing Ag-NPs.

## Figures and Tables

**Table 1:** Characteristics of biochars obtained from different biomasses (*Q*: variable surface charge, *S_BET_*: specific surface area, *V_t_*: total pore volume, *V_m_*: micropore volume, *M*: micropore contribution, *D*: average pore diameter).

Type Biochar	*Q* [cmol/kg]	Carboxylic Groups [cmol/kg]	Lactonic Groups [cmol/kg]	Phenolic Groups [cmol/kg]	*S_BET_* [m^2^/g]	*V_t_* [cm^3^/g]	*V_m_* [cm^3^/g]	*M* [%]	*D* [nm]
**BV**	99.7	26.7	34.9	104.1	98.96	0.049	0.037	75.5	2.02
**BP**	66.7	13.8	9.8	92.3	83.97	0.041	0.034	82.9	1.96
**BT**	48.6	6.9	23.8	20.7	1.92	0.0096	0.0015	15.6	19.94

**Table 2:** Kinetic parameters of Ag-NP adsorption on biochars (*q_e_*-Ag-NP removal capacity at equilibrium; *k*_1_, *k*_2_-reaction rate constants).

	Pseudo First-Order (Lagergren)			Pseudo Second-Order (Ho and Mckay)		
	*k*_1_·10^−2^ [1/min]	*q_e_* [mg/g]	R^2^	*k*_2_·10^−2^ [g/mg·min]	*q_e_* [mg/g]	R^2^
**BV**	2.1	45.2	0.69	0.44	88.9	0.99
**BP**	1.9	41.2	0.51	0.24	75.2	0.99
**BT**	1.5	30.3	0.84	0.10	72.5	0.98

**Table 3:** Isotherms parameters of Ag-NP adsorption on biochars (*K_F_*, *K_L_*, 1/*n*: constants; *Q_m_*: maximum amount of adsorbate in the monomolecular layer).

	Freundlich Isotherm			Langmuir Isotherm		
	*K_F_* [mg/g(L/mg]^1/*n*^	1/*n*	R^2^	*K_L_* [L/mg]	*Q_m_* [mg/g]	R^2^
**BV**	0.46	0.41	0.97	5.98·10^−3^	114.94	0.90
**BP**	1.69·10^−6^	0.72	0.98	2.74·10^−5^	12.97	0.69
**BT**	6.01·10^−9^	0.92	0.98	3.39·10^−7^	4.6	0.95
